# Expectations in the Ultimatum Game: Distinct Effects of Mean and Variance of Expected Offers

**DOI:** 10.3389/fpsyg.2018.00992

**Published:** 2018-07-26

**Authors:** Peter Vavra, Luke J. Chang, Alan G. Sanfey

**Affiliations:** ^1^Behavioral Science Institute, Radboud University, Nijmegen, Netherlands; ^2^Centre for Cognitive Neuroimaging, Donders Institute for Brain, Cognition and Behaviour, Radboud University, Nijmegen, Netherlands; ^3^Department of Functional Neuroplasticity, Medical Faculty, Otto von Guericke University, Magdeburg, Germany; ^4^Psychological and Brain Sciences, Dartmouth College, Hanover, NH, United States

**Keywords:** decision making, social, fairness, expectations, Ultimatum Game

## Abstract

Being treated fairly by others is an important need in everyday life. Experimentally, fairness can be studied using the Ultimatum Game, where the decision to reject a low, but non-zero offer is seen as a way to punish the other player for an unacceptable offer. The canonical explanation of such behavior is inequity aversion: people prefer equal outcomes over personal gains. However, there is abundant evidence that people's decision to reject a low offer can be changed by contextual factors and their emotional state, which cannot be explained by the inequity aversion model. Here, we expand a recent alternative explanation: rejections are driven by deviations from expectations: the larger the difference between the actual offer and the expected offer, the more likely one is to reject the offer. Specifically, we provided participants with explicit information on what kind of offers to expect using histograms depicting distribution of offers given in a previous experiment by the same proposers. Crucially, we showed four different distributions, manipulating both the mean and the variance of these expected sets of offers. We found that 50% of our participants clearly and systematically changed their behavior as a function of their expectations (11% followed the standard-economic model of pure self-interest and 39% where not distinguishable from the inequity-aversion model). Using a logistic mixed-model analysis, we found that the mean and variance differently affect the decision to reject an offer. Specifically, the mean expected offer affected the threshold of what offers are acceptable, while the expected variance of offers changed how strict participants were about this threshold. Together, these results suggest that social expectations have a more complex nature as current theories propose.

## 1. Introduction

Human societies thrive largely due to our ability to construct, and then adhere to, social norms, which can be thought of as shared expectations as to how we should behave toward each other. When people deviate from these norms, we are typically willing to punish transgressors, even if it comes at a personal cost (Fehr and Gächter, 2002). A key question therefore is what motivates us to punish in such situations. The Ultimatum Game (UG) is a well-studied task that has been used to examine such decisions experimentally (Güth et al., 1982). In this simple two-player game, the first player, usually deemed the Proposer, is allocated a sum of money and must make an offer to a second player, termed the Responder, as to how that money should be split. Then, the Responder decides to either accept or reject that offer. If accepted, the money is split as had been proposed; if rejected, however, both players get nothing. Results in this game are highly reliable—importantly, low offers, that is around 10–20% of the total amount, are typically perceived as unfair and are rejected more than half of the time (Camerer, 2003).

From a perspective of pure economic self-interest, it is irrational to reject any non-zero offer, as any amount of money should be preferred to receiving nothing at all. The canonical explanation of why people reject low offers is inequity aversion (Fehr and Schmidt, 1999), which proposes that we monitor the payoffs for ourselves and others, and that any deviation from equality leads to dissatisfaction. Specifically, the inequity aversion account of the Responder's decision to accept or reject an unfair (but non-zero) offer represents this decision as a trade-off between a monetary gain (positive utility) on the one hand, and inequality (negative utility) on the other. Across individuals the relative balance of these two aspects will differ, but once the dissatisfaction of the inequality reaches a certain level, the Responder will forgo the money and reject the unfair proposal (of course leading to no gain for the Proposer as well). Note that inequity aversion can account for some contextual factors which have been shown to affect Responders willingness to accept low offers. For example, after having worked for the right to be the Proposer, players typically make lower offers, and these offers are in turn accepted at a higher rate by the Responder, presumably because people perceive an equitable allocation of money as one that is also proportional to the relative inputs (Hoffman et al., 1996).

However, despite the compelling inequity aversion account, both behavioral (Sanfey, 2009) and neuroimaging studies (Chang and Sanfey, 2013; Xiang et al., 2013) have clearly shown that people also adapt their decisions based on what kind of offers they expect. That is, their expectations of what they will receive can play a large role in their subsequent decisions. For example, when players expect to see low offers, they are in turn more likely to accept these low offers than when their prior expectations were higher. Crucially, this cannot be accounted for by inequity aversion—no change between inputs exists across these scenarios, as the relevant expectations are independent of the actions of the Proposer. Therefore, an alternative explanation has been proposed, namely that instead of deriving disutility from a deviation from equal payoffs, people instead take deviations from prior expectations into account. An initial empirical study of this effect (Sanfey, 2009) demonstrated that participants who had been led to believe that offers would be fair (so-called “high expectations” group) rejected offers at a significantly higher rate than another group of players who had been told that offers would be unfair (“low expectations” group), despite both groups seeing an identical set of offers. Follow-up work extended this finding by examining naturally occurring differences in the expectations of their sample of Responders. By taking the deviations between the actual offer and their participants' prior expectations into account, this substantially better explained decision-making than an inequity aversion account alone (Chang and Sanfey, 2013). These studies demonstrate that players do possess prior knowledge about the type of offers they will likely receive, and importantly, that they use this knowledge of an “average” offer in their subsequent decision-making. Similarly, by altering the average offer from the first half to second half of the experiment, there is evidence of a trial-by-trial reward prediction error, indicating that people track offers and update their expectations based on their prior experience (Xiang et al., 2013). Therefore, it appears that players in the Ultimatum Game have a good awareness of the average offer they can expect, either via experimentally-handed down declarative knowledge, or via their own understanding of the appropriate social norm of fairness, and that this average offer weighs heavily in their accept/reject decisions.

Outside the domain of fairness-related choice, expectations also play a crucial role in financial decisions. For risky financial decisions such as portfolio management, a crucial aspect of the decision-making process is the expected distribution of payoffs. In fact, a common definition of risk is the variance of payoffs around the mean (Rothschild and Stiglitz, 1970; Levy and Levy, 2004). Interestingly, higher-order distributional moments like skew and kurtosis also play a role in risky decision-making (Noussair et al., 2014), suggesting that people evaluate the payoff of risky outcomes by comparing them to the expected distribution of outcomes, not simply the mean or variance. Importantly, many computational formulations (e.g., Bayesian learner) and the associated neural regions (e.g., anterior Insula) seem to be the domain-general, i.e., appertain to risk-taking and social decision making, hinting at the possibility of a common processes for evaluating choice options in light of one's expectations of payoffs. An intriguing possibility therefore is that the social expectations participants both form and use in the Ultimatum Game also may be comprised of more than just the average of the expected set of offers. Indeed, previous work found evidence of a neural variance prediction error, indicating that participants also track how strongly offers vary across a set (Xiang et al., 2013). Though this study did not manipulate both mean and variance independently, this suggests that Responders compare the current offer to the distribution of offers they expect.

**Figure 1:**
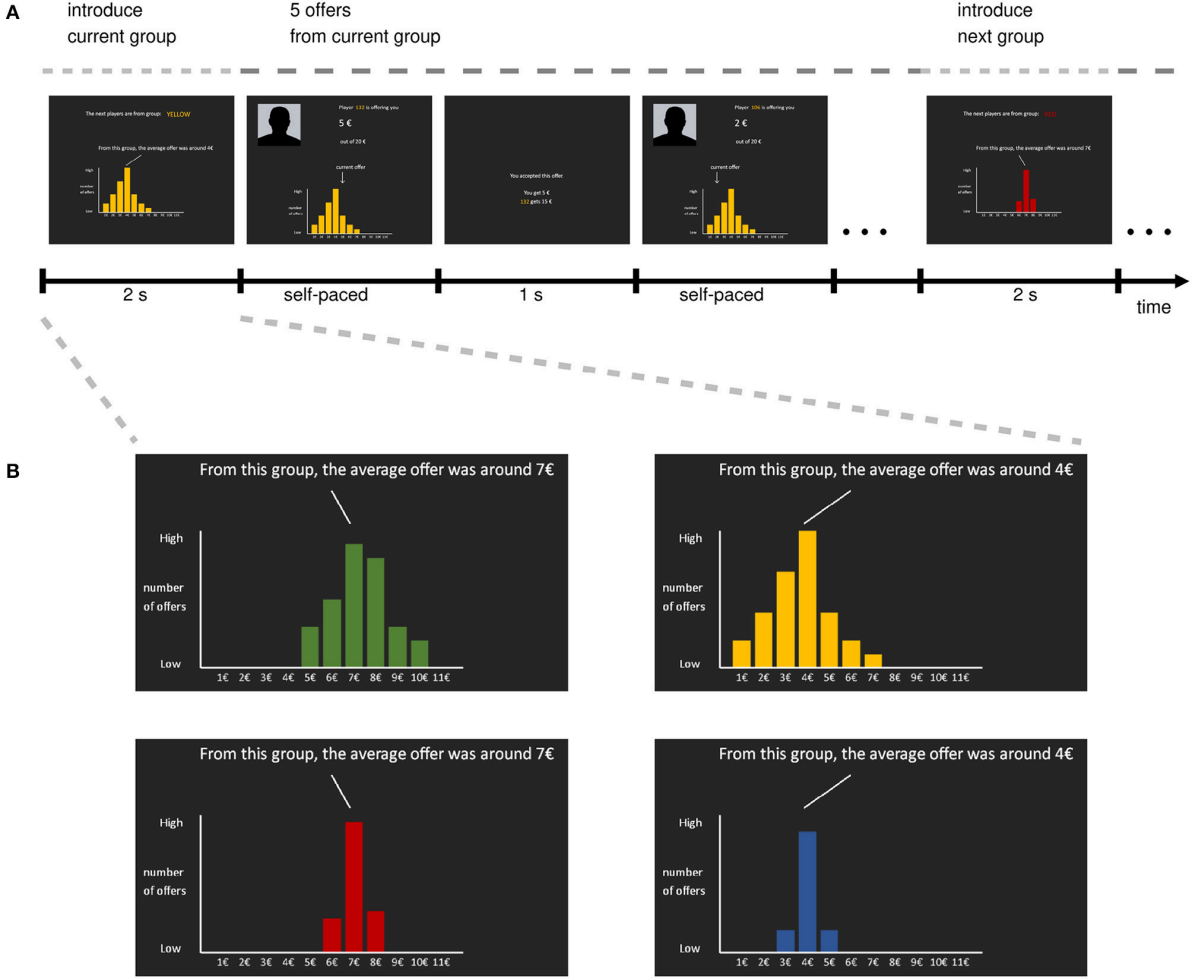
Task structure. **(A)** Time-line of UG task. The four conditions are organized into a mini-block design, where every five rounds a new group of Proposers (i.e., one of the conditions) is randomly picked. At the beginning of each mini-block, the upcoming group is introduced by color and the distribution of offers these Proposers purportedly gave in a previous experiment (i.e., the expectation manipulation). Then participants play five rounds of the UG with five different Proposers from that group with a pot-size of €20. Then, a new mini-block starts by introducing the next group. **(B)** The four groups participants encountered during the task. Importantly, the depicted distributions vary across two dimensions, namely mean and variance, leading to a 2 × 2 within-subject design for the expectations of offers.

In this study, we extended this previous work on expectations in the UG by explicitly investigating whether Responders take into account not only the mean expected offer, as has been shown, but also the particular distribution of the offers. Specifically, in a novel within-subject design (trial structure shown in Figure 1) we manipulated participants' a priori expectations of upcoming offers along two dimensions, the mean and the variance of the distribution of offer: We provided participants with histograms of offers given by the current group of Proposers in a previous experiments. In line with previous studies, we hypothesized that expecting different offers on average would lead to different rejection rates for unfair offers. Additionally, we hypothesized that the variance of offers might have a modulatory effect on this relationship, such that Responders might be less responsive to deviations from the mean expected offer when coming from a high variance set. Finally, we explored whether participants' initial beliefs, that is, what participants expect prior to playing the UG and receiving any specific information on the upcoming groups, also affected their accept/reject decisions.

## 2. Method

### 2.1. Subjects

A total of 62 participants (83 % female, mean age = 22.2 years) completed the study for either course credit or payment (€10). Participants also received a bonus based on their actual performance—a round of the Ultimatum Game (UG) was randomly chosen at the end of the experiment and their monetary outcome was paid out as a bonus to ensure that participants made real decisions (maximum €10). The study protocol was approved by the local ethics committee (CMO region Arnhem-Nijmegen, The Netherlands) under the general ethic approval (CMO 2014/288), and all the experimental methods were conducted in accordance with these guidelines. All participants provided written informed consent in accordance Declaration of Helsinki and the guidelines of local ethics committee.

### 2.2. Initial belief elicitation

After participants familiarized themselves with the rules of the UG, we elicited participants' a priori beliefs about the kind of offers they expected to encounter: For a total pot amount of €20, participants were asked to estimate how many of 100 Proposers would offer €0, €1, €2, €3, ⋯€18, €19, €20. They also indicated what kind of offer they would make themselves.

### 2.3. Ultimatum game & expectation induction

During the UG, participants purportedly played with players from four different groups. Participants were told that the proposers provided offers as part of a previous experiment and would be paid out if participants were to accept their offer and that trial was selected for pay-out. Participants were told that “from previous experiments we know that some groups of people give a lot, while others give a little. You will be playing with people from four different groups. To give you an idea what kind of player you are being paired up with, we will provide you with information about how much these people offered in a previous experiment”. In addition, participants were told that proposers' offers for the current experiment were prerecorded, and would be paid out if participants were to accept their offer and that trial was selected for pay-out. To manipulate participants' expectations, each group was described with a single histogram representing the prior offers of that group (Figure 1). Crucially, the histograms differed on two dimensions: the mean (low/high) and the variance (low/high) of the depicted distribution, leading to a 2 × 2 design. To ensure that participants could interpret the histograms accurately, they were extensively instructed beforehand as to how the histograms were constructed from a set of offers; the distributions were constructed to be slightly asymmetric, as piloting revealed that symmetric distributions were somewhat unrealistic to participants (full instruction material available, see section Data Availability).

Participants played 180 rounds of the UG (45 rounds per condition), each round with a new, unique partner. All UG rounds involved splitting a pot of €20. The offers participants received in each of the four conditions only partially matched the depicted distribution of offers: 30 offers were used to create the histograms and, hence, were a perfect match; to expose participants also to some offers not present in the depicted distributions (i.e., deviations from expectations), the remaining 15 offers where drawn uniformly from the set {€1;€3;€5;€7;€9}. This design was chosen as a compromise between two extremes: One the one hand, if the offer sets were identical across all conditions, it is possible that participants would learn to ignore the histograms by tracking offers and learning that the histograms are unrelated to the actual offer set. On the other hand, if the offer sets matched the histograms perfectly, offers would not deviate from expectations, and hence we would be unable to assess whether deviations from expectations matter. Although this led to an unbalanced design in offer sets across the four conditions, we used these partially matching sets of offers as a trade-off between these two opposing goals.

To further decrease the chance that participants would track the actual set of offers and learn that the histograms are only partially predictive, we shuffled the conditions in a “mini-block” design: Every five trials, one group was randomly chosen, indicated by a screen showing the upcoming group and the associated histogram. This screen also explicitly indicated the mean of that distribution. Then participants played five rounds of the UG with Proposers from this group, before another random group selection was made. Each offer screen also depicted the histogram and indicated the current offer using an arrow (see Figure 1A). This made the relationship of current offer and histogram as salient as possible, and removed any need to keep the distribution in working memory. After participants finished the UG, they were debriefed and paid.

### 2.4. Data analyses

All behavioral statistics were computed using R statistical package (R Core Team, 2017).

We conducted two complementary sets of analyses. First, we modeled the choice data of all participants who rejected at least one offer (*N* = 55) and estimated population effects using a logistic mixed model; second, we classified each participant as following one of three possible choice-models: pure self-interest, inequity-aversion, or the expectation model.

Specifically, we first fitted a logistic mixed model estimating the probability to accept or reject (dependent variable) at each round of the UG. Predictor variables included the offer amount, mean-condition (low vs. high), variance-condition (low vs. high), and all their interactions as fixed effects, and participant as the only random intercept to account for the repeated-measures structure of the data. We centered the offer amount (mean offer €5.33) before estimating the mixed models using lme4 (Bates et al., 2015) to make the fitting process more numerically stable, as suggested in the documentation of lme4. To determine p values, we computed Type 3 Likelihood Ratio Tests as implemented in the mixed function of the package afex (Singmann et al., 2018). We also tried to include random slopes as suggest previously (Barr et al., 2013), but the model did not converge so we report the intercept-only model.

To account for participants' initial beliefs during the UG, for each participant we calculated the mean of the elicited distribution, which quantifies the average offer each participant expects, as well as the standard deviation, which quantifies beliefs about how much the offer amount might vary. We mean-centered both of these variables (again, for more stable estimation using the lme4 package) before adding them as fixed effects to a second mixed-model of the UG decisions.

To gain a more qualitative sense of how prevalent different behaviors are, we classified each participant's behavior as pure-self interest (when they accepted all offers), or as either being motivated by inequity-aversion or expectation-deviation. To achieve this, we estimated two logistic regression models for each participant who rejected at least one offer: The simpler model included only offer amount and an intercept as predictors. This model is a simple reparametrized version of the inequity-aversion model (Fehr and Schmidt, 1999) as no hyperfair (i.e., above half the pot amount) offers were present in our experiment. The second model included predictors indicating the expectations at each trial, namely the mean-condition and variance-condition, as well as all interactions (including with the offer amount), i.e., six additional parameters, which is a reparametrized version of the expectation model, without restricting the direction of the effect of expectations. We assessed whether the more complex expectation model significantly improved the model fit using the Likelihood Ratio Test. This is a rather conservative test, because the two models differ by six parameters, requiring a substantial improvement in model-fit (difference in deviances >12.59) so that a participant's choice-pattern is classified as adhering to the expectation model.

For supplementary analyses of the reaction times, see supplementary materials (Supplementary Data Sheet 1).

### 2.5. Data availability

Data, experimental materials including the instruction materials and analyses are available from the Donders Institute for Brain, Cognition and Behavior repository at (see http://hdl.handle.net/11633/di.dccn.DSC_3014030.01_615).

## 3. Results

**Figure 2:**
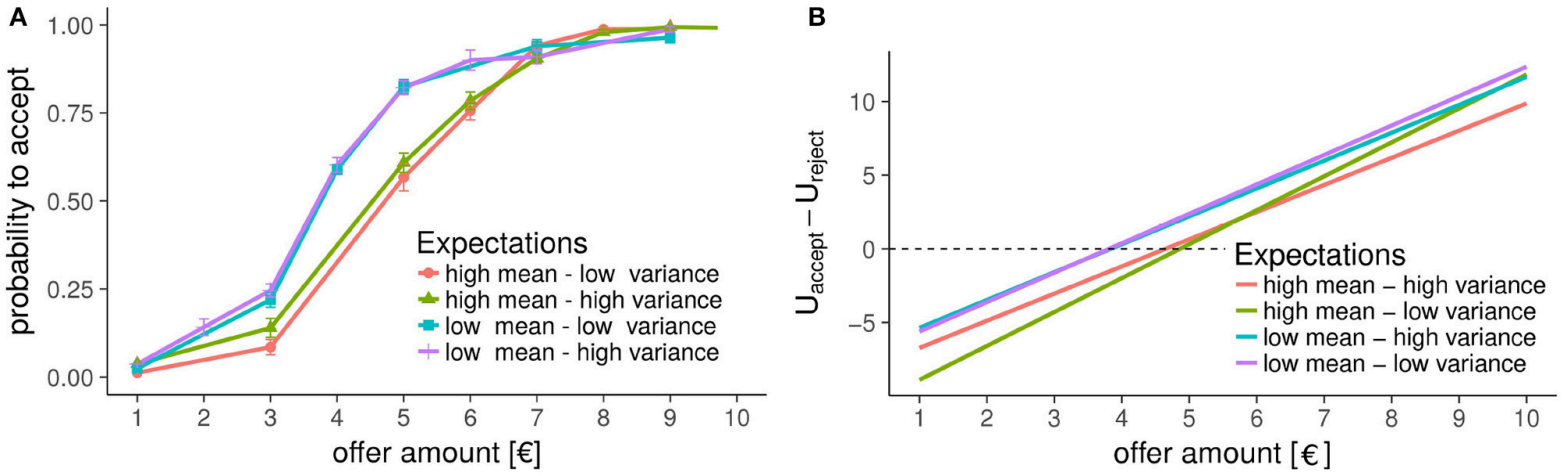
Decision in the Ultimatum Game. **(A)** Between-subject mean probability to accept an offer in the UG. Even without leveraging the within-subject design, it is evident that the mean expected offer substantially changes how likely one is to accept unfair offers (e.g., €5 out of a €20 pot-size). In addition, especially low offers (≤ €3) are accepted more frequently in the high-variance conditions as compared to the respective low-variance conditions. **(B)** The difference in utilities to accept vs. reject a certain offer, as implied by the fitted logistic mixed-model. The effect of mean expected offers is captured by the intercept, whereas the variance changes the slope, which is mostly driven by the difference in slopes in the high-mean conditions.

In the UG, as expected, participants rejected low offers more often than high offers [main effect of the centered offer amount, *Estimate* = 2.01(0.05), *OR* = 7.45, $\chi_{\left(1\right)}^{2}=5901.3$, *p* < 2.210^−16^]. In addition to this canonical observation, our expectation manipulation also influenced participants' decisions: When expecting low offers, participants were more likely to accept all offers [main effect of mean-condition, *Estimate* = −0.88(0.06), *OR* = 0.17, $\chi_{\left(1\right)}^{2}=236.5$, *p* = 5.3*10^−5^]. Additionally, the variability of the expected offers impacted how strongly participants' decisions were affected by the offer amount [interaction effect of variance and offer-amount, *Estimate* = −0.14(0.04), *OR* = 0.75, $\chi_{\left(1\right)}^{2}=16.33$, *p* = 0.013; three-way interaction of offer amount, variance-condition, and mean-condition, *Estimate* = −0.09(0.04), *OR* = 0.70, $\chi_{\left(1\right)}^{2}=6.227$, *p* = 0.01] such that participants rejected fewer low offers when they expected high variability as compared to when they expected more consistency across, and more so when expecting the average offer to be high (fitting the same model logistic mixed model to the low and high mean conditions separately shows that the interaction of offer-amount with the variance reaches significance only in the high mean conditions). Together, these effects indicate that participants were more likely to accept low offers of €3 when they were expecting low offers, and when they expected offers to vary to a greater extent (see Figure 2). No other effects were significant. When performing the above analyses on subsets of the data one for the high mean and one for the low mean conditions—we see the following pattern: in the high mean conditions, there is a significant effect of variance [*Estimate* = −0.20(0.05); *OR* = 0.66; $\chi_{\left(1\right)}^{2}=19.85$; *p* < 0.10^−5^]; in the low mean conditions, this effect is not significant although in the same direction [*Estimate* = −0.063(0.05); *OR* = 0.88; $\chi_{\left(1\right)}^{2}=1.43$; *p* = 0.225].

**Figure 3:**
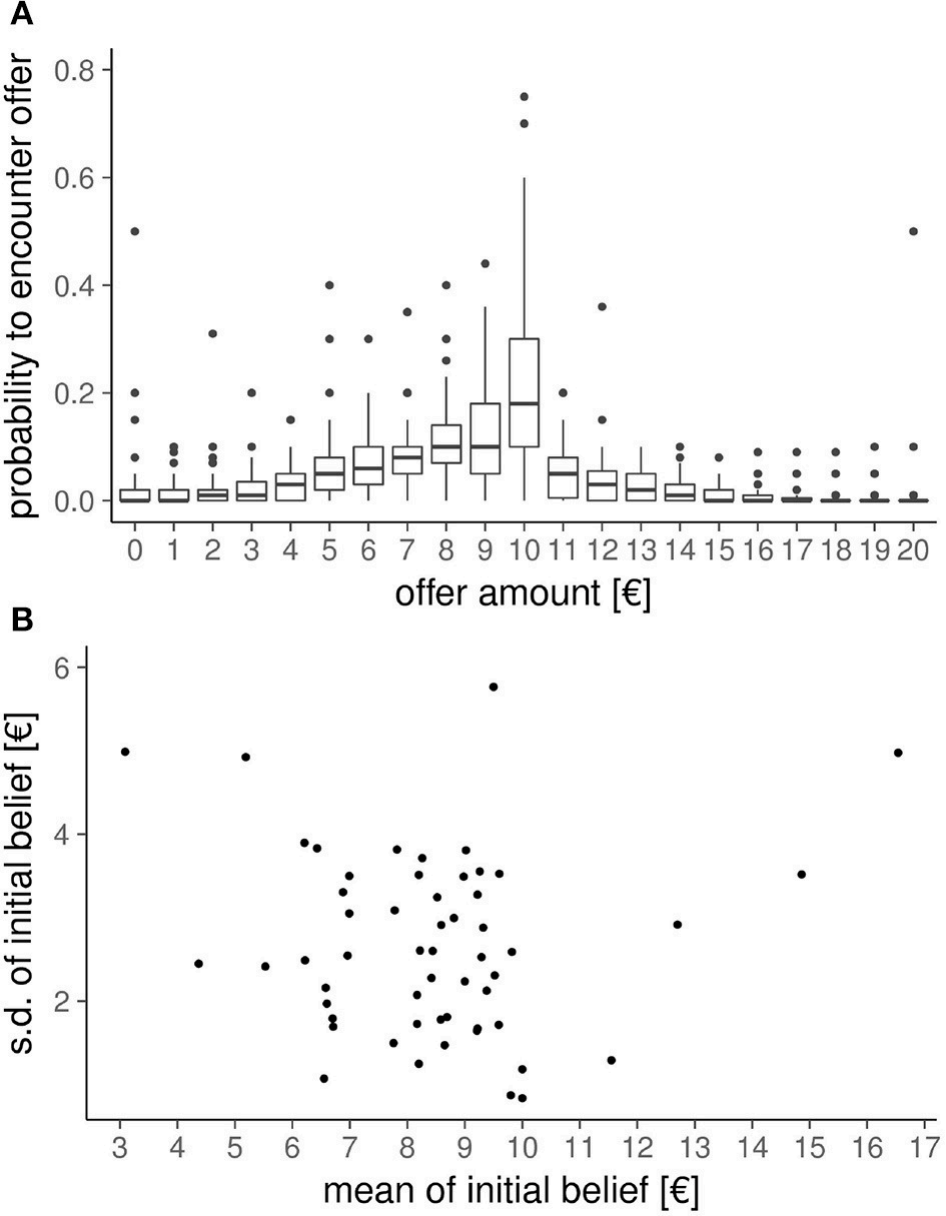
Initial beliefs. Before playing the Ultimatum Game and receiving any information on the groups participants would encounter during the experiment, we elicited for each participant their initial beliefs about how likely they would encounter each possible offer amount. **(A)** Between-subject distribution of initial beliefs. **(B)** Between-subject variability in mean and standard-deviation of the initial belief distribution of each participant. The substantial variability in these two aspects of the initial beliefs (as well as their low correlation of *r* = 0.008) allows as to quantify their influence on the UG decisions.

Before playing the UG and receiving any of information on the upcoming group of Proposers, participants indicated their initial beliefs about what distribution of offers they expect. The between-subject mean of these initial beliefs are shown in Figure 3A. On average, participants expected an offer of €8.5 from a €20 pot. If the elicited belief distributions differ across participants, then the impact of these initial beliefs on the UG decisions can be quantified. To summarize each participant's initial beliefs, we calculated the mean and standard deviation of the belief distribution. The between-subjects variability of the mean of the belief distribution and the standard deviation of the belief distribution is shown in Figure 3B. The variance of each of these aspects (s.d. of mean of initial beliefs *s*_*mean*_ = €2.19, standard deviation of standard deviation of initial beliefs *s*_*s*.*d*._ = €1.09) as well as their negligible correlation (*r* = 0.008) suggest that we can include both of these variables into the mixed-model.

To assess the influence of the initial beliefs on their decisions in the UG, we first added the centered mean and centered standard deviation of the initial expected offers as fixed effects, without adding any interaction-terms, which is analogous to performing an ANCOVA-like analysis for logistic regression, i.e., “controlling for” these two variables. This did not change any of the aforementioned significant effects, and the fixed effects of mean and variance of initial expectation were not significant. However, when including all higher-order interactions with offer-amount, mean-condition, and variance-condition, we found additional significant effects. Specifically, this revealed an additional significant interaction between the mean and variance manipulations [*Estimate* = 0.12(0.06), *OR* = 1.62, $\chi_{\left(1\right)}^{2}=3.95$, *p* = 0.047] which qualifies the aforementioned effect of the mean-condition in such a way that participants accepted all offers more often when they expected to get low offers, but this effect was stronger when they did not expect the offers to vary much.

Interestingly, we also found that differences in initial beliefs were related to the magnitude of the effect-sizes for of the effects described above: The mean of the initial beliefs showed significant interactions with the offer amount [*Estimate* = −0.24(0.03), *OR* = 0.79, $\chi_{\left(1\right)}^{2}=58.0$, *p* < 10^−4^] and mean-condition [*Estimate* = 0.13(0.04), *OR* = 1.30, $\chi_{\left(1\right)}^{2}=11.5$, *p* = 0.0007], and the three-way interaction with offer amount and variance-condition [*Estimate* = 0.05(0.02), *OR* = 1.11, $\chi_{\left(1\right)}^{2}=4.45$, *p* = 0.035]. Importantly, these interactions are modulations of effects described above—without taking the initial beliefs into account—and the direction of the interactions is consistently such that the magnitude of the effects decreases for participants who had an initial belief of receiving high offers. The standard deviations of the initial beliefs also showed significant interaction effects, namely, with offer amount [*Estimate* = −0.34(0.04), *OR* = 0.71, $\chi_{\left(1\right)}^{2}=54.3$, *p* < 10^−4^], mean-condition [*Estimate* = 0.42(0.06), *OR* = 2.32, $\chi_{\left(1\right)}^{2}=56.2$, *p* < 10^−4^], variance-condition [*Estimate* = 0.11(0.05), *OR* = 1.25, $\chi_{\left(1\right)}^{2}=4.42$, *p* = 0.036]. Finally, there were also interaction effects between mean and variance of prior expected offers and the manipulations: the three-way interaction with offer-amount [*Estimate* = 0.08(0.02), *OR* = 1.08, $\chi_{\left(1\right)}^{2}=24.94$, *p* < 10^−4^], the 3-way interaction with mean-condition [*Estimate* = −0.07(0.02), *OR* = 0.87, $\chi_{\left(1\right)}^{2}=10.16$, *p* = 0.0014].

More qualitatively, we found that 11% (7/62) of the participants in our sample accepted all offers—in line with a standard economic model of pure self-interest—while the majority of participants reject at least some offers. Crucially, 50% (31/62) of the participants were classified as clearly following the expectation model, while only 39% (24/62) seemed not to take expectations into account above chance level, in line with a simpler inequity-aversion motivation.

## 4. Discussion

In this study, we explored how expectations about upcoming offers in the Ultimatum Game affected participants' decisions to either accept or reject a given offer. In a novel within-subject design, we provided participants with differing expectations about sets of upcoming offer amounts by showing four different histograms, each depicting the distribution of offers that a particular group had previously made in a prior experimental setting. Across these four distributions of offers, we manipulated both the mean, and the variance of the respective offer set. Participants in our study were therefore provided with four different sets of expectations—high mean offer with low variance, high mean offer with high variance, low mean offer with low variance, and low mean offer with high variance. Importantly, the provision of these expectations should theoretically not actually impact the participants' decision, as all relevant information is provided in the actual offer itself. That is, what has been “expected” is, or should be, largely irrelevant to the decision to accept or reject a particular monetary offer. Nonetheless, we clearly demonstrated that both mean and variance of the depicted distributions impacted participants' Ultimatum Game choices, underlining, and extending, the importance of expectations in social decision-making. Crucially, the two aspects of the distributions that were experimentally manipulated had a different effect on the accept/reject decisions. Expecting high offers decreased the likelihood of accepting offers, while expecting greater variation in offers increased the likelihood of accepting offers, low offers in particular. When controlling for participants initial beliefs, we also found that in the high variance conditions people were willing to accept lower offers more often than in the low variance conditions, especially when expecting high offers. Therefore, these results show that expecting a certain type of offer impacts decision-making. Importantly, we extend knowledge about how expectations can alter choice by showing that both the mean and the variance of those expectations plays an important role in decision-making.

To gain a more complete understanding as to how expectations impact our social choices, we also elicited participants' initial beliefs about the offers they might see in the game, prior to the experiment and thus before seeing any of the histograms. Participants differed in both the offers they initially believed they might receive, as well as how varied a set of offers would likely be. We found that participants who had relatively high initial beliefs about offer amounts and were, thus, presumably disappointed by the set of relatively unfair offers actually encountered during the experiment, then rejected low offers more often. However, if they also believed that Proposers would vary more in their offers this effect was smaller, in that they accepted low offers more often. Importantly, this pattern is consistent with the effects of the handed-down expectations. Although these initial beliefs were not the focus of the present study, we believe that many factors influencing participants' accept/reject decisions can potentially be accounted for by changes in people's expectations. For example, the finding that Proposers offer less (in relative terms) and Responders more often accept these smaller offers when the stakes are large (Andersen et al., 2011) might be due to both players having different expectations of what kind offers are likely to be given (namely lower ones). Therefore, we believe a fruitful avenue for future research is to study which factors influence participants initial beliefs about the kind of offers they are likely to receive. Importantly, our results suggest that these investigations should go beyond simply asking participants what kind of “average” offer they expect to see and instead elicit a full distribution of the likelihood of all offers.

Interestingly, we also found interaction effects between participants' initial beliefs and the expectations that were handed down in the course of the experiment. We did not have specific hypotheses for these interactions, so these analyses were exploratory. Nonetheless, we would like to highlight that almost all significant interactions of the initial beliefs and the expectations involved the effects of our manipulations described above—without accounting for the initial beliefs. This suggests that participants built up subjective expectations based on both their initial beliefs and the information provided prior to the trials, and then used these subjective expectations as the basis for their accept/reject decisions. The exact nature of how these different expectations are integrated into one coherent expected distribution of offers is an interesting question for future research and beyond the scope this study. One possibility is a Bayes-rule-like updating, where the initial belief forms a prior, the handed-down expectations the likelihood, and the resulting subjective expectations would be the posterior distribution of the offers, similar to the trial-by-trial updating of expectations shown before (Xiang et al., 2013).

Importantly, the results outlined here are difficult to account for via the standard inequity aversion model, which proposes that decisions to reject low amounts are driven by comparing the current offer to the equitable state of receiving half of the pot (Fehr and Gächter, 2002). While half of our participants either accepted all offers or appeared to conform to the aforementioned inequity aversion predictions, the other half clearly exhibited behavior that could not be accounted for by either a purely self-interested or an inequity averse strategy: these set of participants' behavior was well accounted for by an expectation account. More specifically, the Fehr-Schmidt inequity aversion model (Fehr and Schmidt, 1999) specifies that any deviation from an equal split will be a source of disutility, and as such does not accommodate changes in this level of disutility due to different expectations. While of course the inequity aversion model could be modified by adding a new expectation parameter to define what “equity” is, changing the model in this way also changes its fundamental nature. This is because we believe the underlying psychological mechanism is markedly different between the two accounts. An intrinsic assumption of the inequity aversion model is that there is one fair allocation of resources, given certain inputs by the different players (namely a proportional one). In the case of a standard Ultimatum Game, these inputs are equal and thus an equal split of the money is the equitable and fair allocation. However, when expectations are taken into account, a different notion of what is fair is possible: People evaluate a given allocation by comparing it to their expectations (Chang and Sanfey, 2013; Sanfey et al., 2014). If, with identical inputs, two different contexts are associated with different expectations (e.g., because of what happened before in those contexts, as in our case), there will be different notions of what is fair. Put differently, there is no single fair allocation, when considering only inputs and outputs; instead fairness depends on the context and other “variables” which go beyond a simple balance of inputs and outputs. Our findings replicate, and extend, recent research demonstrating that prior expectations can impact player's decisions, often in surprisingly strong ways.

The current study extends the expectations model by demonstrating that, in addition to the mean amount, the variance of the expected set of offers impacts decisions: expectations of the variance of an offer set changes how strongly any deviation from expectations is weighted. There are two different ways this effect of variance can be interpreted. One is that the expected variance of the offer set could signal certainty about one's expectation. This is in a similar vein to perceptual decision-making (e.g., Pouget et al., 2013) where variance is a result of noise around the stimulus and therefore makes estimation of the mean (true) signal more difficult. Accordingly, when a group of people behave in a heterogeneous manner, it is naturally more difficult to learn the average behavior of that group. However, given that no learning was necessary in our experimental design, as participants explicitly observed the entire distribution of offers in a histogram format, this explanation is, we believe, less likely. Unlike the perceptual process, the average offer here does not necessarily represent the “true” behavior of a particular group—people may simply vary in how they behave.

A second, and more intriguing, possibility is that social expectations are complex entities that consist, amongst other things, of distributional information such as how homogeneously a group behaves. Indeed, in everyday life, we can imagine situations with different associated expectations. In one situation we might expect to be treated nicely by everyone, whereas in other situations we might expect people to behave more variable, some being nice, some not so much. We can also imagine situations where we would expect most people to be nice, but occasionally encountering a few “bad apples.” From this perspective, we may not just hold expectations about the average or most likely behavior, but we may also explicitly track higher order features of that distribution, such as variability (homogeneity of a group) and skew (bad apples). Indeed, when comparing initial beliefs across different participants, the average offer participants initially thought they would encounter did not correlate with their belief about how much people might vary. Based on this interpretation, one prediction therefore is that higher moments of the distribution, such as skew and kurtosis, are also being taken into account when evaluating the offer at hand in the Ultimatum Game. If this is the case, the decision to accept or reject an offer may have similarities to risky decision-making where higher-order moments have been shown to play a role (Noussair et al., 2014). It is worthwhile pointing out, however, that in contrast to risky decision-making where variance in expectations is due to the probabilistic nature of the outcomes (e.g., a sure option of €5 vs. a 50/50 chance of winning €10 or receiving nothing), the decision to accept or reject in the UG is of course not probabilistic: participants know with complete certainty the consequences of both choice options.

Although the focus of our experiment was to compare the inequity-aversion (Fehr and Schmidt, 1999) model with the expectation-deviation model (Chang and Sanfey, 2013; Battigalli et al., 2015), there are several mechanisms proposed to underly the rejection of low offers. These views are typically seen as falling into two camps (see e.g., Karagonlar and Kuhlman, 2013). On the one hand, people are proposed as altruistically punishing low offers to enforce a social fairness norm (Rabin, 1993; Fehr and Gächter, 2002; Fehr and Fischbacher, 2003). Indeed, people are willing to sacrifice their own money even if they are not the target of the unfairness (Fehr and Fischbacher, 2004). On the other hand, the low offer itself can be seen as an insult by the responder—the proposer would give a low offer only if they believe the responder is likely to accept it—causing a negative emotional response. This more self-centered emotional response, in turn, could drive the ensuing rejection(Pillutla and Murnighan, 1996). In fact, people are willing to reject unfair offers even in variants of the game where such a decision does not affect the outcomes of the proposer (Impunity Game) (Yamagishi et al., 2009)—likely ruling out a motivation to enforce a social norm. Instead, people seem to want to send a message: they do not want to be part of an unfair interaction. From the perspective of the expectation-deviation model, however, these views are not necessarily opposing: the average expected offer indicates a context-dependent social norm and any offer below that will cause a negative emotional response. This will be the case independently of who the target of the allocation is, accounting for rejections of unfair offers on behalf of others; once there is a deviation, people are likely to reject these low offers to feel better, explaining why people reject offers even in the Impunity Game. Previous research has suggested that individual differences like social value orientation affect how strong the emotional response to an unfair offer is, leading to differences in rejection rates independent of the mean expected offer—even when people rate the low offers as equally unfair, suggesting they differ in how strictly they apply social norms (Karagonlar and Kuhlman, 2013). The results of our experiment add to this view by highlighting that another aspect of expectations, namely the expected variability of offers, influences how strict the current social norm is in a context-dependent manner.

The Ultimatum Game has been widely used to investigate people's evaluation of, and response to, fairness. Here, we demonstrated that the fundamental decision as to whether a certain allocation of money is evaluated as either acceptably fair or not depends to an important degree on people's prior expectations of what kind of offer they will receive. We show that different aspects of these expectations affects assessment of fairness in different ways: expecting higher offers raises the bar for what is acceptable, while expecting high variability leads to greater flexibility in applying a criterion of fairness. Overall, this is consistent with the possibility that social expectations are complex entities, consisting of distributional information about how likely one is to encounter different types of behavior from interaction partners, and that these set of expectations form the basis of our fairness judgments.

## Author contributions

PV, LC, and AS designed the experiment. PV collected and analyzed the data. PV, LC, and AS wrote the manuscript.

### Conflict of interest statement

The authors declare that the research was conducted in the absence of any commercial or financial relationships that could be construed as a potential conflict of interest.

## References

[B1] Andersen S. Ertaç S. Gneezy U. Hoffman M. List J. A. (2011). Stakes matter in ultimatum games. Am. Econ. Rev. 101, 3427–3439. 10.1257/aer.101.7.3427

[B2] Barr D. J. Levy R. Scheepers C. Tily H. J. (2013). Random effects structure for confirmatory hypothesis testing: keep it maximal. J. Mem. Lang. 68, 255–278. 10.1016/j.jml.2012.11.001 24403724 PMC3881361

[B3] Bates D. Mächler M. Bolker B. Walker S. (2015). Fitting linear mixed-effects models using lme4. J. Stat. Softw. 67, 1–48. 10.18637/jss.v067.i01

[B4] Battigalli P. Dufwenberg M. Smith A. (2015). Frustration and anger in games, in CESifo Working Paper Series No. 5258. Available at SSRN: https://ssrn.com/abstract=2591839 (Accessed June 10, 2018).

[B5] Camerer C. F. (2003). Behavioral Game Theory: Experiments in strategic interaction. Princeton, NJ:Princeton University Press.

[B6] Chang L. J. and Sanfey, A. G. (2013). Great expectations: neural computations underlying the use of social norms in decision-making. Soc. Cogn. Affect. Neurosci. 8, 277–284. 10.1093/scan/nsr094 22198968 PMC3594719

[B7] Fehr E. and Fischbacher, U. (2003). The nature of human altruism. Nature 425, 785–791. 10.1038/nature02043 14574401

[B8] Fehr E. and Fischbacher, U. (2004). Third-party punishment and social norms. Evol. Hum. Behav. 25, 63–87. 10.1016/S1090-5138(04)00005-4

[B9] Fehr E. and Gächter, S. (2002). Altruistic punishment in humans. Nature 415, 137–140. 10.1038/415137a 11805825

[B10] Fehr E. and Schmidt, K. M. (1999). A theory of fairness, competition, and cooperation. Q. J. Econ. 114, 817–868.

[B11] Güth W. Schmittberger R. Schwarze B. (1982). An experimental analysis of ultimatum bargaining. J. Econ. Behav. Organ. 3, 367–388.

[B12] Hoffman E. McCabe K. A. Smith V. L. (1996). On expectations and the monetary stakes in ultimatum games. Int. J. Game Theor. 25, 289–301.

[B13] Karagonlar G. and Kuhlman, D. M. (2013). The role of social value orientation in response to an unfair offer in the ultimatum game. Organ. Behav. Hum. Decis. Process. 120, 228–239. 10.1016/j.obhdp.2012.07.006

[B14] Levy H. and Levy, M. (2004). Prospect theory and mean-variance analysis. Rev. Financ. Stud. 17, 1015–1041. 10.1093/rfs/hhg062

[B15] Noussair C. N. Trautmann S. T. Van De Kuilen G. (2014). Higher order risk attitudes, demographics, and financial decisions. Rev. Econ. Stud. 81, 325–355. 10.1093/restud/rdt032

[B16] Pillutla M. M. and Murnighan, J. K. (1996). Unfairness, anger, and spite: emotional rejections of ultimatum offers. Organ. Behav. Hum. Decis. Process. 68, 208–224.

[B17] Pouget A. Beck J. M. Ma W. J. Latham P. E. (2013). Probabilistic brains: knowns and unknowns. Nat. Neurosci. 16, 1170–1178. 10.1038/nn.3495 23955561 PMC4487650

[B18] R Core Team (2017). R: A Language and Environment for Statistical Computing. Vienna: R Foundation for Statistical Computing.

[B19] Rabin M. (1993). Incorporating fairness into game theory and economics. Am. Econ. Rev. 83, 1281–1302.

[B20] Rothschild M. and Stiglitz, J. E. (1970). Increasing risk: I. a definition. J. Econ. Theor. 2, 225–243.

[B21] Sanfey A. G. (2009). Expectations and social decision-making: biasing effects of prior knowledge on Ultimatum responses. Mind Soc. 8, 93–107. 10.1007/s11299-009-0053-6

[B22] Sanfey A. G. Stallen M. Chang L. J. (2014). Norms and expectations in social decision-making. Trends Cogn. Sci. 18, 172–174. 10.1016/j.tics.2014.01.011 24582437

[B23] Singmann H. Bolker B. Westfall J. Aust F. (2018). afex: Analysis of Factorial Experiments. R package version 0.20-2. https://CRAN.R-project.org/package=afex

[B24] Xiang T. Lohrenz T. Montague P. R. (2013). Computational substrates of norms and their violations during social exchange. J. Neurosci. 33, 1099–1108. 10.1523/JNEUROSCI.1642-12.2013 23325247 PMC3631781

[B25] Yamagishi T. Horita Y. Takagishi H. Shinada M. Tanida S. Cook K. S. (2009). The private rejection of unfair offers and emotional commitment. Proc. Natl. Acad. Sci. U.S.A. 106, 11520–11523. 10.1073/pnas.0900636106 19564602 PMC2703666

