# Supplementary material for: Expectations in the Ultimatum Game: Distinct Effects of Mean and Variance of Expected Offers
Source: Front Psychol. 2018 Jul 26;9:992. doi: 10.3389/fpsyg.2018.00992 (PMC6070732; doi:10.3389/fpsyg.2018.00992)
Supplement: Supplementary file 1 [file Data_Sheet_1.docx]

Supplementary Material

Expectations in the Ultimatum Game: Distinct Effects of Mean and Variance of Expected Offers

Peter Vavra*, Luke J. Chang, Alan G. Sanfey

*** Correspondence:** Corresponding Author: p.vavra@donders.ru.nl

# Reaction Times Analysis

Whether participants accept or reject a certain offer depended on their expectation of the average offer and how varied they expect offers to be. Thus, it is interesting to see whether there are any systematic differences in reaction times (RTs) as a function of expectations.

Supplementary Fig. 1 shows the mean RTs for all offer amounts and in the four conditions present in our data.


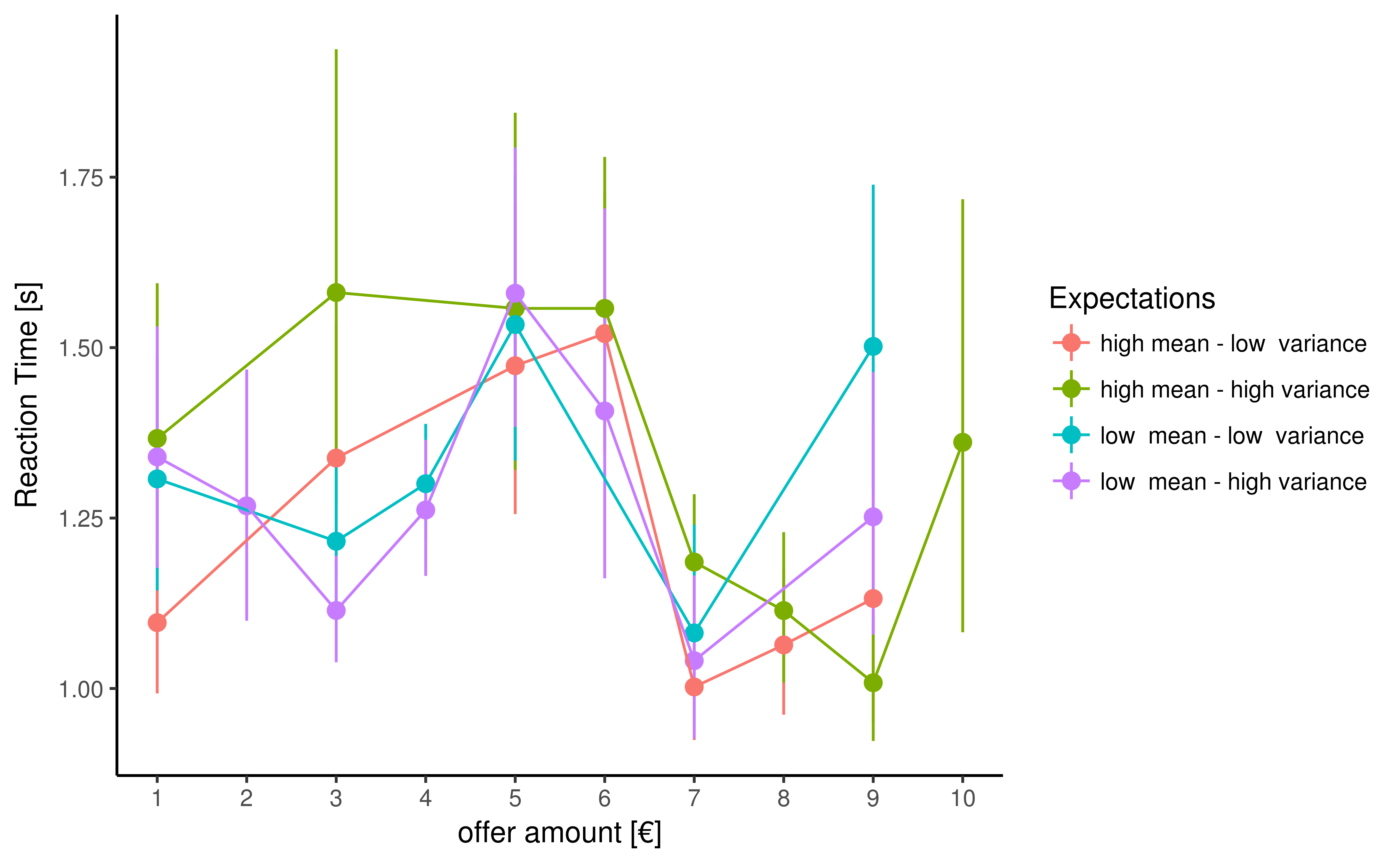


**Supplementary Figure 1.** Mean reaction times for the decision to accept or reject an offer (out of a pot of 20€). Vertical lines indicate bootstrapped 95% confidence intervals of the mean.

To statistically assess whether there are any systematic differences as a function of expectations, we modeled RTs as a function of the 2 (low vs high expected mean offer) x 2 (low vs high expected variance of offers) design using a linear mixed model.

Specifically, we limited our analysis to offers from the set {1; 3; 5; 7; 9} as only these offers where present in all four conditions (see Method section). Further, we modeled RTs for each offer amount separately, resulting in five linear mixed models. As dependent variable we chose the log-transformed RTs to account for the non-normal distribution of reaction times. As independent variables we chose the full 2x2 design of mean, variance (coded as -0.5 and 0.5 so that the estimated parameters can directly be interpreted as a difference in RTs between the two levels of the predictor) and their interaction term and we included a random intercept for each subject to account for the within-subject nature of the task. We fitted the models using the *mixed* function in the *afex* package which in turn calls the *lmer* function in the *lme4* package. We estimated *p*-values using parametric bootstrapping.

Supplementary Table 1 shows estimated model parameters for all offer amounts. When deciding to accept or reject an offer of 3€, participants were significantly slower when they expected high offers on average (main effect of mean expected offer: b = 0.128; *p* = .03). We also observed differences when deciding about higher, more fair offers. Specifically, when deciding about an offer of 7€, participants were significantly slower when they expected the offer to be high on average and offers to vary more (interaction effect of mean and variance: b = 0.202; *p* = .008). More interestingly, we found that when deciding about offers that are almost half the pot-amount, namely offers of 9€ – which are almost always accepted – participants decided significantly more slowly when they expected offers to be low on average (main effect of mean expected offer: b = -0.218; *p* = .001) and when they expected offers to be consistently around that mean offer (main effect of variance: b = -0.095; *p* = .04).

| **Offer amount** | **Effect of mean (p-value)** | **Effect of variance (p-value)** | **Interaction of mean and variance (p-value)** |
| --- | --- | --- | --- |
| € 1 | -0.064 (.23) | 0.041 (.40) | 0.107 (.20) |
| € 3 | 0.128 (.03) | -0.008 (.87) | 0.124 (.20) |
| € 5 | 0.037 (.54) | -0.004 (.92) | -0.108 (.24) |
| € 7 | -0.053 (.21) | 0.058 (.14) | 0.202 (.008) |
| € 9 | -0.218 (.001) | -0.095 (.04) | 0.063 (.52) |

**Supplementary Table 1:** Estimated effect sizes on Reaction Times (parenthesis: bootstrapped p-value) for five offers amounts.
